# Broad and Complex Roles of NBR1-Mediated Selective Autophagy in Plant Stress Responses

**DOI:** 10.3390/cells9122562

**Published:** 2020-11-30

**Authors:** Yan Zhang, Zhixiang Chen

**Affiliations:** 1 Department of Landscape and Horticulture, Ecology College, Lishui University, Lishui 323000, Zhejiang, China; yzhang@lsu.edu.cn; 2 Department of Botany and Plant Pathology and Purdue Center for Plant Biology, 915 W. State Street, Purdue University, West Lafayette, IN 47907-2054, USA

**Keywords:** autophagy, NBR1, plant stress responses, selective autophagy receptor, protein aggregates, plant heat tolerance, plant virus interaction

## Abstract

Selective autophagy is a highly regulated degradation pathway for the removal of specific damaged or unwanted cellular components and organelles such as protein aggregates. Cargo selectivity in selective autophagy relies on the action of cargo receptors and adaptors. In mammalian cells, two structurally related proteins p62 and NBR1 act as cargo receptors for selective autophagy of ubiquitinated proteins including aggregation-prone proteins in aggrephagy. Plant NBR1 is the structural and functional homolog of mammalian p62 and NBR1. Since its first reports almost ten years ago, plant NBR1 has been well established to function as a cargo receptor for selective autophagy of stress-induced protein aggregates and play an important role in plant responses to a broad spectrum of stress conditions including heat, salt and drought. Over the past several years, important progress has been made in the discovery of specific cargo proteins of plant NBR1 and their roles in the regulation of plant heat stress memory, plant-viral interaction and special protein secretion. There is also new evidence for a possible role of NBR1 in stress-induced pexophagy, sulfur nutrient responses and abscisic acid signaling. In this review, we summarize these progresses and discuss the potential significance of NBR1-mediated selective autophagy in broad plant responses to both biotic and abiotic stresses.

## 1. Introduction

Autophagy is an evolutionarily conserved process in eukaryotic organisms for degradation of cytoplasmic constituents including proteins and organelles in the lysosomes or vacuoles [1]. There are at least three types of autophagy, known as macroautophagy, chaperone-mediated autophagy and microautophagy. Macroautophagy is the pathway that has been most extensively characterized and is often referred to simply as autophagy [1]. Autophagy is usually induced in response to a variety of physiological and environmental stimuli and plays an important role in cell homeostasis under unfavorable and pathological conditions including starvation, extreme temperature, aging and pathogen infection [2]. Induction of autophagy is initiated by the formation of an isolation membrane called phagophore that can expand to capture cytoplasmic components and enclose the sequestered cargos to form a separate compartment, the double-membrane autophagosome [3,4]. The autophagosome is then primed to fuse with the lysosomes or vacuoles for degradation of captured materials by lysosomal/vacuolar hydrolases. Autophagosome biogenesis is a complex membrane process that requires more than 40 autophagy-related (ATG) proteins. These ATG proteins function in several physiologically continuous, but mechanistically distinct steps and are organized as several functional groups, which include (i) the ATG1–ATG13–ATG17 scaffold, formed upon activation of the ATG1 kinase during the early stage of autophagy induction, (ii) the class III phosphatidylinositol 3-kinase (PtdIns3K)–ATG14–ATG6 (Beclin 1) complex for the nucleation and assembly of the initial phagophore membrane, and iii) two interrelated ubiquitin-like conjugation systems, ATG12–ATG5–ATG16 and ATG8–PE (phosphatidylethanolamine), which are required for the regulation of membrane elongation and expansion of the forming autophagosomes [1].

The core process of autophagy and ATG proteins are highly conserved in all eukaryotic organisms including plants [1,5,6,7]. Using genetic and molecular approaches, extensive studies over the past two decades or so have demonstrated an important role of autophagy in almost all aspects of plant life, particularly in plant stress responses [5,8,9]. Autophagosome biogenesis and ATG gene expression are both induced by diverse abiotic stress conditions including nutrient starvation, heat, salt, drought and oxidative stresses [10,11,12,13,14,15]. Autophagy mutants and transgenic silencing lines are hypersensitive to nutrient starvation and compromised in tolerance to these abiotic stresses [10,11,12,13,14,15]. In addition, autophagy plays an important role in plant innate immunity. Plant mutants or transgenic silencing lines for autophagy are altered in responses to virulent and avirulent biotrophic pathogens including pathogen-induced hypersensitive responses [16,17,18,19,20,21]. Autophagy deficient mutants are compromised in resistance to necrotrophic pathogens [17,22]. As will be discussed below, autophagy is also involved in plant interaction with viral pathogens through such mechanisms as regulation of antiviral RNA silencing and targeting degradation of viral proteins. Autophagy also plays important roles in plant growth and development including root growth, leaf senescence, pollen and endosperm development [8,20,23,24,25].

Even though autophagy was initially considered to be a nonselective process of bulk degradation of intracellular contents, it has been now well established that the broad roles of autophagy are primarily mediated by selective clearance of specific cellular structures [26,27,28,29]. Ubiquitin-like ATG8, which is required for phagophore initiation, elongation and maturation, also plays a critical role in selective autophagy [27]. Upon attachment of the lipid PE to its carboxyl terminus through a conjugation pathway, ATG8 is anchored in both the inner and outer membrane of autophagosomes and provides a docking platform for the selective recruitment of cargos through selective autophagy receptors [27]. Most selective autophagy receptors recognize specific cargos and also interact with membrane-anchored ATG8 through ATG8-interacting motifs (AIMs), which have the W/Y/F-X-X-L/I/V consensus core sequence [27]. The AIM motif binds a hydrophobic patch on ATG8 known as AIM docking site [27]. Recently, a new class of selective autophagy receptors have been identified, which interact with ATG8 through ubiquitin-interacting motif (UIM)-like sequences for high-affinity binding to an ATG8 interaction site different from the AIM docking site [30,31]. Through assays with candidate UIM-containing proteins and unbiased screens, a large number of UIM-based ATG8 interactors have been identified in plants, yeast, and humans [32]. Discovery of the new class of selective autophagy receptors greatly expands the scope of selective autophagy [32].

A large number of autophagy receptors from nonplant organisms have been identified that mediate the selective autophagic degradation of a wide range of cargoes including protein aggregates, signaling complexes, mitochondria, peroxisomes, endoplasmic reticulum (ER), ribosomes and pathogens [27,29,33,34]. In plants, a substantial number of autophagy receptors have also been identified, characterized and functionally analyzed. These plant autophagy receptors include *Arabidopsis* protein TSPO (outer membrane tryptophan-rich sensory protein), an ATG8-interacting heme-binding protein that targets the degradation of porphyrins through autophagy [35]. TSPO also targets the aquaporin PIP2;7 for degradation in the vacuole to reduce its levels to regulate water permeability under conditions of heat and drought stress [36,37]. ATG8-interacting 1 (ATI1) and 2 (ATI2) proteins are closely related autophagy receptors partially associated with the ER under normal conditions but mainly associated with a different type of spherical compartments under carbon starvation [38,39]. ATI1 is also found on bodies associated with plastids under carbon starvation and is involved in autophagy-dependent trafficking of plastid proteins to the vacuole [39]. The three related ATI3 autophagy receptors from *Arabidopsis* play a critical role in plant heat tolerance and resistance to the necrotrophic fungal pathogens at least in part through interaction with ER-localized UBAC2 proteins and mediating selective autophagy of specific unknown ER components [40]. *Arabidopsis* DSK2 acts as a selective autophagy receptor that targets BR1-EMS suppressor 1(BES1) to modulate brassinosteroid signaling and stress tolerance [41]. *Arabidopsis* ORM1 and 2 proteins mediate selective autophagy of pattern recognition receptor FLS2 to negatively regulate FLS2-mediated defense [42]. More recently, *Arabidopsis* cytosolic protein C53 has been identified as a receptor for selective autophagy of certain ER domains (ER-phagy) [43]. C53 is also an ER-phagy receptor in mammalian cells [44]. The proteasome subunit RPN10 is a selective autophagy receptor that mediates selective autophagy of the 26S proteasome when the proteasome is inhibited chemically [31]. RPN10 interacts with ATG8 through its UIM-like sequence and is increasingly associated with the proteasome when it is ubiquitinated [30].

While many selective autophagy receptors identified in plants are plant-specific, others are evolutionarily conserved and have homologs in nonplant organisms. Plant NBR1 is structurally related to human NBR1 and SQSTM1/p62 selective autophagy receptors that act in aggrephagy [14,45,46]. Since their first reports almost ten years ago [45,46], a number of studies have revealed an important role of NBR1 in plant responses to a broad spectrum of stress conditions including heat, salt, drought and oxidative stress [14,15,47]. More recently, new discoveries have been made in the understanding the roles and mode of action of NBR1 in the modulation of plant heat stress memory, plant-viral interaction and other physiological processes associated with plant stress responses. In this review, we discuss the structure, evolution and roles of plant NBR1 in responses to a broad spectrum of biotic and abiotic stresses. We also discuss the molecular mechanisms by which NBR1-mediated selective autophagy modulates plant stress responses by highlighting the cargo proteins recognized and targeted by NBR1 and how their turnover leads to altered plant stress responses.

## 2. Structure, Evolution and Function of NBR1

Plant NBR1 is a structural homolog and functional hybrid of mammalian autophagy receptors NBR1 and p62, which differ in size but share similar domains and important features [45,46,48,49]. Both mammalian p62 and NBR1 proteins contain an N-terminal PB1 (Phox and Bem1p) domain followed by a ZZ-type zinc finger domain, an LC3-interacting region or LIR motif (also known as AIM motif in yeast) and a C-terminal UBA (ubiquitin-associated) domain [49]. As in many other selective autophagy receptors, the LIR motifs mediate direct interaction with ATG8, while the C-terminal UBA domain recognizes mono- and polyubiquitin. The PB1 domain of p62 mediates polymerization of p62, but the related human NBR1 protein lacks the most N-terminal basic charge cluster and, therefore, cannot polymerize through its PB1 domain [49]. However, human NBR1 can form heterodimers with p62 via its PB1 and can self-interacts via a coiled coil domain [49]. Mammalian p62 and NBR1 function as selective autophagy receptors for autophagic degradation of protein aggregates (aggrephagy), mitochondria (mitophagy), peroxisomes (pexophagy), endoplasmic reticulum (reticulophagy) and pathogens (xenophagy) [50]. p62 and NBR1 also have noncanonical role in signaling independent of autophagy [51,52,53,54]. For example, p62 promotes the expression of inflammatory genes via NF-κB through TRAF6 binding by its TRAF6-binding (TB) domain [53]. p62 can also activate mTORC1, which can upregulate c-Myc [55]. These functions are independent of the UBA or LC3-interacting region (LIR) domains of p62, which are required for its role as an autophagy receptor [53].

**Figure 1:**
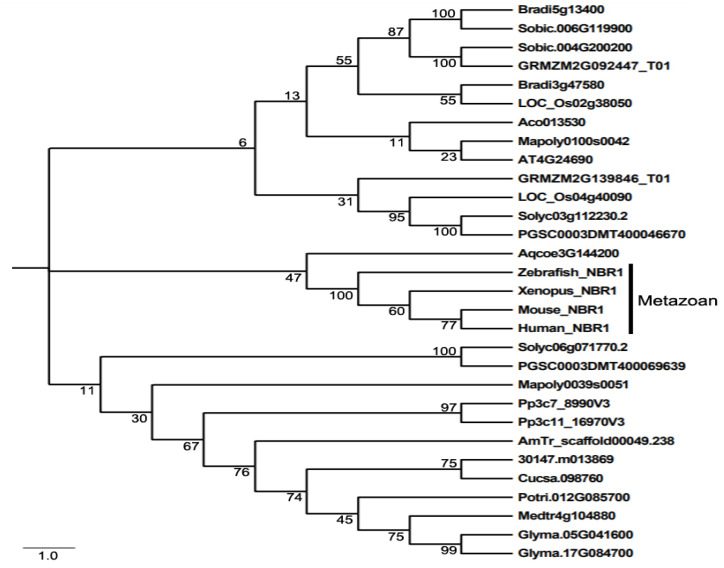
The phylogenetic relationship of NBR1 homologs from plants and metazoans. The tree was inferred using the neighbor-joining method. Phylogenetic analyses were conducted in MEGA5. Bootstrap values from 1000 replicates were used to assess the robustness of the tree. NBR1 homologs in the phylogenetic analysis include those from *Homo sapiens* (Human NBR1), *Mus musculus* (Mouse NBR1), *Danio rerio* (Zebrafish NBR1), *Xenopus tropicalis* (Xenopus NBR1), *Marchantia polymorpha* (Mapoly0100s0042 and Mapoly0039s0051), *Physcomitrella patens* (Pp3c7_8990V3 and Pp3c11_16970V3), *Amborella trichopoda* (AmTr scaffold00049.238), *Ananas comosus*(Aco013530), *Brachypodium distachyon* (Bradi3g47580 and Bradi5g13400), *Oryza sativa* (LOC_Os02g38050 and LOC_Os04g40090), *Sorghum bicolor* (Sobic.004G200200 and Sobic.006G119900), *Zea mays* (GRMZM2G139846_T01 and GRMZM2G092447_T01), *Aquilegia coerulea* (Aqcoe3G144200), *Solanum lycopersicum* (Solyc03g112230.2 and Solyc06g071770.2), *Solanum tuberosum* (PGSC0003DMT400069639 and PGSC0003DMT400046670), *Populus trichocarpa* (Potri.012G085700), *Ricinus communis* (30147.m013869), *Arabidopsis thaliana* (AT4G24690), *Cucumis sativus* (Cucsa.098760), *Glycine max* (Glyma.05G041600 and Glyma.17G084700), *Medicago truncatula* (Medtr4g104880).

Even though NBR1 has a domain architecture similar to that of p62, it has a highly conserved globular domain characterized by the presence of 4 highly conserved tryptophan (W) residues that is absent in p62 [49]. Using this four tryptophan or FW domain to distinguish p62 and NBR1 homologs through the eukaryotic kingdom, it was discovered that only metazoans contain both p62 and NBR1 homologs, while most nonmetazonan organisms have a single NBR1 homolog [49]. Plant NBR1 homologs lack the coiled coil domain of mammalian NBR1 but have two C-terminal UBA domains [45]. In model plant *Arabidopsis*, there is a single gene encoding an NBR1 homolog. Like p62, *Arabidopsis* NBR1 can homo-oligomerizes through the N-terminal PB1 domain, indicating the plant NBR1 share some functional properties with p62 [45]. In addition, only the C-terminal UBA domain of the two UBA domains of *Arabidopsis* NBR1 binds ubiquitin. We have searched the sequenced genomes of representative plants along the evolutionary tree for genes encoding NBR1 homologs and identified 27 NBR1-encoding genes from 2 spore-bearing and 15 seed plants (Figure 1). As previously reported, some plants contain a single gene encoding NBR1. However, most plants including the spreading earthmoss (*Physcomitrella patens*), tomato, potato, rice, maize, purple false brome (*Brachypodium distachyon*) and soybean contain two genes encoding NBR1 proteins (Figure 1). Maize, soybean and purple false brome are known to be polyploid plants that have gone genome duplications during their evolutionary history [56,57,58], which could account for the presence of more than one NBR1 genes in their genomes. To analyze the evolutionary relationship of the conserved protein family, we performed phylogenetic analysis of NBR1 homologs from 17 plant and 4 animal species. As shown in Figure 1, there are three major clades in the phylogenetic tree. All the NBR1 proteins from the animals clustered in one clade, which intriguingly also include the NPR1 from *Aquilegia coerulea*, while those from plants clustered in two separate clades (Figure 1). These results indicate that the topology of phylogenetic tree for NBR1 homologs from animals and plants is in general agreement with the evolutionary tree of the organisms. However, there is no clear separation of clustering of NBR1 homologs between spore-bearing and seed plants and likewise, NBR1 proteins from monocot and dicot plants do not clustered separately in the clades of seed plants (Figure 1). Furthermore, while the twin NBR1 homologs from some plants such as soybean and P. patens clustered together, other twin NBR1 homologs from the same plant species including tomato, potato, rice, maize and purple false brome do not group together in the tree (Figure 1), indicating significant sequence divergence between these twin homologs. The evolutionary significance of the sequence variation of the NBR1 homologs is unclear but could reflect potential functional diversification of the two NBR1 homologs in these plants.

A number of groups have reported functional genetic analysis of plant NBR1 through characterization of *nbr1* mutants or transgenic silencing lines [14,15,46,47,59,60,61,62]. *Arabidopsis nbr1* knockout mutants grow and develop normally under normal growth conditions and is not essential for general autophagy, or for the selective clearance of peroxisomes, mitochondria, or the ER [14,60,62]. Plant NBR1 is also dispensable in age- and darkness-induced senescence but may modulates senescence under certain conditions such as short-day growth condition [14,59]. The *Arabidopsis nbr1* mutants are also normal in resistance to a necrotrophic pathogen [14]. However, the *nbr1* mutants are compromised in plant tolerance to heat, oxidative, salt, and drought stresses [14,47]. The role of NBR1 in plant abiotic stress tolerance is dependent on its interaction with ATG8. In *Arabidopsis*, NBR1 also reduces growth of bacterial pathogen *Pseudomonas syringae* by suppressing the establishment of an aqueous extracellular space (‘water-soaking’) [61]. Therefore, the *nbr1* mutants display some but not all of the phenotypes of autophagy-deficient mutants. As will be discussed below, more recent studies have revealed specific cargo proteins of plant NBR1 and their roles in the modulation of plant heat stress memory, plant-viral interaction, senescence, reactive oxygen species-induced pexophagy and nutrient responses. 

## 3. NBR1 in Heat Tolerance and Heat Stress Memory

**Figure 2:**
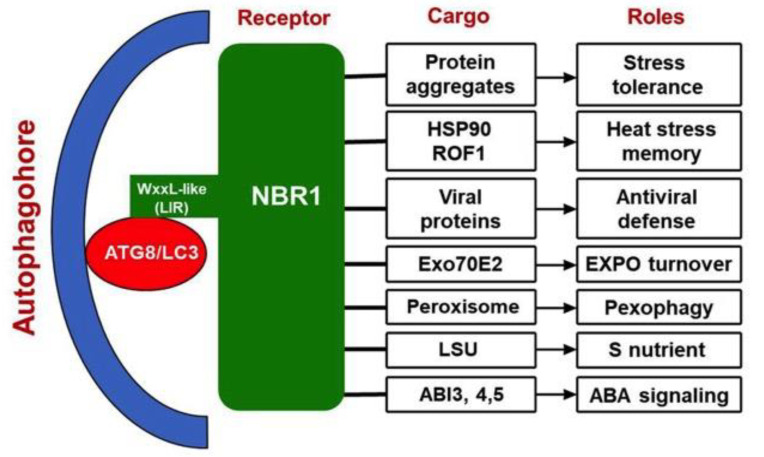
NBR1-mediated selective autophagy in plant stress responses. The cargo proteins and biological roles of NBR1 in aggrephagy, heat stress memory, antiviral defense and Exo70E2 are well supported by biochemical, molecular and genetic evidence. Studies through analysis of protein localization, gene expression, protein-protein interactions and transgenic overexpression have also suggested possible roles of NBR1 in stress-induced pexophagy, S nutrient response and ABA signaling.

Heat stress caused by high temperature is a fundamental threat to all living organisms including plants. High temperature causes protein misfolding and misfolded proteins are highly toxic and must be efficiently removed to reduce cellular proteotoxic stress [63,64,65]. Selective autophagy functions in protein quality control by targeting degradation of misfolded and other nonnative proteins and therefore also plays an important role in plant response to heat stresses [14,15,40,47]. *Arabidopsis* mutants and transgenic silencing tomato plants for ATG5, ATG7 and NBR1 are all compromised in heat tolerance, associated with increased development of heat stress symptoms and compromised photosynthesis in heat-stressed leaf tissues [14,15,40,47]. The compromised heat tolerance of *atg5*, *atg7*, and *nbr1* mutants was also associated with elevated accumulation of insoluble, detergent-resistant proteins that were also highly ubiquitinated under heat stress [14,15,40,47]. NBR1 also accumulated to high levels with an increasing enrichment in the insoluble protein fraction in the autophagy-deficient mutants under heat stress [14,15,40,47]. Through microscopic and biochemical analysis of *nbr1* mutants expressing autophagy markers and an aggregation-prone reporter, it was found that NBR1 is required for the heat-induced formation of autophagic vesicles [56]. Moreover, cytoplasmic puncta containing aggregation-prone proteins, which were rarely observed in wild-type plants, were found to accumulate in *nbr1* mutants under both control and heat stress conditions [56]. Co-localization of NBR1 with the cytoplasmic puncta suggests that *Arabidopsis* NBR1 is a plant aggrephagy receptor and NBR1-mediated autophagy targets ubiquitinated protein aggregates most likely derived from denatured and otherwise damaged nonnative proteins and protein aggregates generated under heat stress (Figure 2).

In animals, the carboxyl terminus of the Hsc70-interacting protein (CHIP) is chaperone-dependent E3 ubiquitin ligase [66,67,68,69]. CHIP plays a critical role in protein quality control by ubiquitinating Hsp70-bound misfolded proteins [66,67,68,69]. CHIP acts as both an Hsp70 co-chaperone through its N-terminal tetratricopeptide repeat (TPR) domain and an E3 ubiquitin ligase through the C-terminal U-box domain [66,67,68,69]. If chaperone-assisted refolding of denatured or damaged proteins fails, CHIP E3 ubiquitin ligase can introduce ubiquitination and thereby target denatured and damaged proteins for degradation by both UPS and autophagy [66,67,68,69]. *Arabidopsis* CHIP is highly similar to animal CHIPs with three tetratricopeptide repeats at the N-terminal side and a U-box domain at the C-terminal side [70,71,72]. To determine the role of CHIP in plant heat tolerance through ubiquitination of misfolded proteins and protein aggregates we have previously reported analysis of CHIP for its coordinated role with the NBR1 autophagy receptor in plant heat tolerance [47]. Two *chip* T-DNA insertion mutants are normal under normal growth conditions but are hypersensitive to heat, salt and oxidative stresses [47]. More importantly, the *chip nbr1* double mutants were more sensitive to heat stress than the *nbr1* and chip single mutants, indicating an additive role of CHIP and NBR1 in plant stress responses [47]. Proteomic profiling of stress-induced protein aggregates is consistent with CHIP and NBR1 mediating two distinct but complementary anti-proteotoxic pathways in plant stress responses [47]. More importantly, stress-induced protein aggregates were still ubiquitinated in the chip mutants [47], suggesting that other ubiquitin E3 ligases are responsible for the ubiquitination of heat-induced protein aggregates targeted by NBR1-mediated selective autophagy.

Very often, plants can be subjected repeatedly to adverse environmental conditions such as high temperature. Once a stress condition ends, plants need to orchestrate the balance between growth recovery and keeping stress memory for better survival when a second and harsher stress occurs later. Heat stress memory and recovery are complex processes regulated at multiple levels, including chromatin remodeling, transcriptional activation of heat-induced genes, regulated turnover of transcripts and proteins important for protein quality control [73,74,75]. Autophagy also helps reset cellular heat stress memory in plants [76]. Autophagy is induced in plants by moderate heat stress and remains highly active long after end of moderate heat stress [76]. The activated autophagy targets specific heat shock proteins for degradation at later stages of the thermorecovery phase [76]. Reduced levels of heat shock proteins lead to reduced heat stress memory, and consequently increased accumulation of protein aggregates after the second heat stress and a compromised heat tolerance [76]. In autophagy-deficient mutants, turnover of induced heat shock proteins is reduced, leading to improved heat stress memory and heat tolerance to the subsequent heat stress [76].

Further analysis has demonstrated that NBR1-mediated selective autophagy plays a critical role in the recovery from heat stress through reduction of heat stress memory [77]. The heat shock transcription factor HSFA2 is a key component of heat stress memory through its role in the induction of heat shock genes [78]. HSFA2 interacts with HSP90.1, a major regulator of heat tolerance [79]. *Arabidopsis* HSP90.1 interacts with ROF1/AFKBP62 (rotamase FKBP 1), a plant homolog of mammalianFKBP4/FKBP52, and regulates plant heat stress responses [79]. The HSP90.1-ROF1 complex remains in the cytoplasm under normal conditions but binds HSFA2 and translocates to the nucleus following exposure to heat stress [79]. Formation of this complex is probably required for enhanced transcriptional activity of HSFA2 and sustained HSP synthesis during heat stress recovery to improve plant response to an imminent recurrence of heat stress [79]. Immunoblot analysis and confocal microscopy revealed that levels of the NBR1 protein, NBR1-labeled puncta, and NBR1 activity are all induced during the heat stress recovery [77]. Co-immunoprecipitation analysis and comparative proteomic analysis identified almost 60 proteins as potential novel targets of NBR1, among which are HSP90.1 and ROF1 [77]. NBR1 mediates the degradation of HSP90.1 and ROF1 by autophagy, which attenuates the expression of heat shock protein genes regulated by the HSFA2 transcription factor, thereby repressing the response to heat stress [73] (Figure 2). In the *nbr1* loss-of-function mutants, there is a stronger heat stress memory [77]. These results indicate that NBR1-mediated selective autophagy regulates plant response to recurrent heat stress. Therefore, the roles of NBR1 in plant responses to heat stress are complex. NBR1-mediated selective autophagy targets protein aggregates generated under heat stress to promote basal plant heat tolerance but also can targets degradation of protein chaperones during the recovery to weaken acquired heat tolerance in plants.

## 4. NBR1 in Plant-Virus Interactions

Autophagy is critical player in animal innate immunity for its role in protecting cells from diverse intracellular pathogens including viruses [80,81,82,83]. As an endolysosomal delivery system, autophagy can promote antiviral response through the transfer of viruses from the cytoplasm to the lysosome for degradation or the transfer of viral components to specific subcellular compartments for the activation of innate or adaptive antiviral immunity [80,81,82,83]. Likewise, autophagy plays an important role in plant antiviral defense. In resistant tobacco plants, silencing of genes for autophagy including ATG3 and ATG7 leads to increased spread of hypersensitive cell death and elevated accumulation of tobacco mosaic virus [18]. In these autophagy-silenced tobacco plants, the virus and viral RNA are both confined only to the infection site in the plants, indicating that the increase in TMV accumulation is not due to increased spread [18]. More likely, plant autophagy limits virus replication and/or movement through degradation of viruses in vacuoles or through effects on other antiviral mechanisms [18].

Double stranded (ds) RNA-induced RNA silencing is an important antiviral mechanism in both animal and plants. Autophagy can also modulate plant antiviral defense by targeting degradation of plant and viral proteins associated with dsRNA-induced RNA silencing. Plant antiviral RNA silencing involves the processing of viral dsRNA into small viral RNAs by the enzyme Dicer. Viral-derived small RNAs guide the degradation of viral RNAs by the RNA-induced silencing complex (RISC). To counter the antiviral defense mechanism, viruses have evolved viral suppressors that inhibit the virus-induced RNA silencing process [84]. P0, an F-box ubiquitin E3 ligase and a viral suppressor of RNA silencing from polerovirus, ubiquitinates the autophagic degradation of ARGONAUTE 1 (AGO1), a key component of RISC that binds to small interfering RNA (siRNA) and carries the RNA slicer activity [85]. Ubiquitinated AGO1 is degraded by selective autophagy instead of the 26S proteasome system based on the sensitivity of degradation to both 3-methyladenine (3-MA), an inhibitor of autophagosome formation, and E64d, a cysteine protease inhibitor of the degradation of autophagic cargo inside autolysosomes, but not to the inhibition of the proteasome [85]. In another study, it was shown that a tobacco calmodulin-like protein (rgs-CaM) functions as an antiviral factor through two distinct antiviral mechanisms, one of which is linked with autophagy [86]. First, rgs-CaM is an interacting protein of HC-Pro and structurally unrelated 2b, two RNA silencing suppressors from tobacco etch virus (TEV) and cucumber mosaic virus (CMV), respectively [86]. The tobacco rgs-CaM protein binds to the dsRNA-binding domains of the RNA silencing suppressors and sequesters them from inhibiting RNA interference (RNAi) [86]. Second, chemical inhibition or suppression of autophagy through gene silencing increased the protein levels of endogenous rgs-CaM and interacting viral RNA silencing suppressors [86]. Furthermore, accumulated endogenous rgs-CaM and interacting viral RNA silencing suppressors were associated with LysoTracker-stained autophagosomes, suggesting that they formed complexes are recruited into autophagosomes for degradation [86]. Thus, tobacco rgs-CaM binds viral RNA silencing suppressors to both decrease the suppressor activity and target their degradation by autophagy [86].

In a more recent study with cauliflower mosaic virus (CaMV), it has been shown that autophagy and NBR1-mediated selective autophagy in particular have complex roles, both anti- and proviral, in the compatible interaction of the dsDNA pararetrovirus with the model plant *Arabidopsis* [87]. First, NBR1-mediated selective autophagy targets nonassembled and virus particle-forming capsid proteins for degradation, thereby restricting the establishment of CaMV infection [87]. As a counter mechanism, the CaMV-induced virus factory inclusions protects capsid proteins against autophagic destruction by sequestering the viral proteins and coordinating particle assembly and storage [87]. Second, virus-triggered autophagy plays a critical role in reducing extensive senescence and tissue death of infected plants. This survival function significantly extends the timespan of virus production, thereby increasing the chances for virus particle acquisition by aphid vectors and CaMV transmission [87]. This role of autophagy is not dependent on NBR1 [87]. These results demonstrate the integration of selective autophagy into plant immunity against viruses and the potential viral strategies to evade and adapt autophagic processes for successful infection (Figure 2).

In another recently reported study, selective autophagy was identified as antiviral pathway during plant infection with turnip mosaic virus (TuMV), a positive-stranded RNA potyvirus [88]. The autophagy cargo receptor NBR1 suppresses viral accumulation by targeting the viral RNA silencing suppressor helper-component proteinase (HCpro), presumably in association with virus-induced RNA granules [88]. Again, the virus has evolved mechanisms to suppress the antiviral mechanism. TuMV seems to antagonize NBR1-dependent autophagy during infection by the activity of distinct viral proteins, thereby limiting its antiviral capacity. As found with CaMV discussed above, NBR1-independent autophagy prevents premature plant death to extend the lifespan of virus reservoirs and particle production [88]. Taken together, these results again illustrate the conserved role of selective autophagy in antiviral immunity and reveal the evolvement of viral protein functions to inhibit autophagy processes (Figure 2).

## 5. NBR1 in the Autophagic Degradation of Exocyst Subunit Exo70E2

As compared to endocytosis, exocytosis is a process that transport materials from inside the cell to the external part of the cell [89]. The exocyst complex directs the secretory vesicles of exocytosis from the Golgi complex to specific locations on the plasma membrane and to mediate their tethering and localization to the membrane immediately before fusion [89]. In *Arabidopsis*, there is a double-membrane organelle termed the exocyst-positive organelle (EXPO), which may be involved in mediating unconventional protein secretion in plants [90,91]. *Arabidopsis*, an exocyst complex subunit, is a marker for EXOP [90]. Upon induction of autophagy, Exo70E2-GFP positive EXPOs and autophagosome were colocalized and delivered to vacuoles for degradation in transgenic *Arabidopsis* plants [90]. Recently, through multiple approaches, it has been demonstrated that *Arabidopsis* NBR1 is a selective receptor for Exo70E2 during autophagy in *Arabidopsis* [59]. First, biochemical and recruitment assays demonstrated that AtNBR1 specifically interacted and recruited Exo70E2 or its EXPO to AtATG8-positive autophagosomes [59]. This interaction of NBR1 with Exo70E2 is not dependent on the UBA domains of NBR1 [59]. Second, in the *nbr1* mutants, the vacuolar delivery of AtExo70E2 or EXPO upon autophagic induction was significantly reduced when compared to that in wild-type plants [59]. Taken together, these results indicate that *Arabidopsis* NBR1-mediated selective autophagy pathway is involved in the vacuolar delivery of Exo70E2 or EXPO in plant autophagy (Figure 2). The physiological significance of NBR1-mediated degradation of Exo70E2 or EXPO is not clear. The exocyst components are known to be involved in autophagy in yeast and mammalian cells [92,93,94]. Exocysts may also play roles in autophagy in plants [95,96]. This notion is supported by the observation that the majority of AtExo70 homologs contain ATG8-interacting motifs and *Arabidopsis* Exo70B1 is required for autophagy-mediated delivery of anthocyanin to the vacuole in *Arabidopsis* [95,96]. In addition, NBR1-mediated selective autophagy may be involved in the regulation of special protein secretion pathway through modulation of the levels of exocyst subunit proteins and the number of EXPO. It would be interesting to determine whether other exocyst complex subunits are also targeted by selective autophagy and, if so, how they are recruited to autophagosomes and what is the physiological significance for their targeted degradation in plants.

## 6. NBR1 in Cadmium-Induced Pexophagy

In mammalian cells, NBR1 is necessary and sufficient for selective autophagic degradation of peroxisomes (pexophagy) [33]. On the other hand, p62 is not required for pexophagy when NBR1 is in excess but its binding to NBR1 increases the efficiency of NBR1-mediated pexophagy [33]. Several reported studies, however, have shown that plant NBR1 is not required for the selective clearance of peroxisomes [60,62]. In the *Arabidopsis* mutants with defective LON2/At5g47040, a protease implicated in peroxisomal quality control, there is reduced responsiveness to the peroxisomally-metabolized auxin precursor indole-3-butyric acid (IBA), heightened degradation of several peroxisomal matrix proteins, and impaired processing of proteins harboring N-terminal peroxisomal targeting signals [62]. Autophagy deficiency ameliorates these defects [62]. Through comparison of peroxisome abundance between the *lon2* single and *lon2* nbr1 double mutants, it was established that NBR1 is not necessary for autophagy of *lon2* peroxisomes [62]. Furthermore, NBR1 overexpression is not sufficient to trigger autophagy of seedling peroxisomes [62]. These results support that *Arabidopsis* targets peroxisomes for autophagic degradation through an NBR1-independent mechanism.

Intriguingly, another recently reported study has implicated NBR1 in heavy metal cadmium (Cd)-induced, reactive oxygen species (ROS)-dependent pexophagy in *Arabidopsis* [97]. Cd treatment induces transient peroxisome proliferation in *Arabidopsis* leaves, which is associated with upregulation of the transcript levels of ATG8 genes and ATG8 proteins [97]. The Cd-dependent induction of pexophagy was demonstrated by the accumulation of peroxisomes in *Arabidopsis* knockout mutants *atg5* and *atg7* [97]. Importantly, ATG8a colocalizes with NBR1 in the electron-dense peroxisomal core, suggesting that NBR1 may be an autophagic receptor for peroxisomes [97]. Protein carbonylation and peroxisomal redox state suggest that protein oxidation may trigger Cd-induced pexophagy [97]. Therefore, while plant NBR1 is not essential for pexophagy, it could play a role in modulating Cd-induced selective autophagy of perophagy (Figure 2). Further studies using molecular genetic approaches will be necessary to confirm the proposed role of plant NBR1 in pexophagy.

## 7. NBR1 in Plant Responses to Sulfur Deprivation

Sulfur (S) is an essential mineral nutrient for plants [98,99]. The plant response to sulfur deficiency includes extensive changes at multiple levels including transcriptome, proteome, metabolome even before the onset of the first visible symptoms of sulfur starvation [100,101,102]. In *Arabidopsis*, members of the plant-specific LSU (response to Low SUlfur) gene family are induced by sulfur deficiency [103]. Although the precise function of LSU-like proteins is still unknown, they have been identified as important stress-related hubs in plant responses to sulfur deficiency [103]. Interestingly, LSU proteins from *Arabidopsis* interact with CAT2 and NBR1, suggesting a possible role of NBR1 in plant S nutrient responses [103] (Figure 2). Consistent with this possibility, plants exposed to S deficit induced autophagy and the elevated transcription of NBR1, suggesting an increased demand for NBR1 during the activation of autophagy under the conditions of S deficiency [104]. Indeed, transcriptome analysis has revealed that NBR1 overexpression altered plant gene expression in response to the low S conditions [104]. One of the NBR1-regulated gene encodes RPS3, which also interacts with NBR1 in a manner independent of the UBA domains of NBR1 [104]. The interaction of NBR1 with a S deficiency-regulated ribosome protein suggests a possible role of NBR1 in ribosomes remodeling in response to S starvation. Furthermore, *Arabidopsis* seedling overexpressing NBR1 have significantly shorter roots than wild type when grown under S deficient conditions in the presence of TOR kinase inhibitors [104]. Thus, overexpression of NBR1 increases sensitivity to inhibition of TOR kinase under S starvation condition, both of which induce autophagy [104]. Apparently, excessive autophagy induction by the additive effect of NBR1 overexpression, starvation, and TOR inhibition cause detrimental effects on *Arabidopsis* growth.

## 8. NBR1 in Abscisic Acid (ABA) Signaling

ABA is a plant hormone that regulates a wide range of cellular and molecular processes in plant growth, development and response to both biotic and abiotic stresses [105,106]. A recently reported study has provided lines of evidence for a role of NBR1 in ABA signaling [107]. First, transcriptomic analysis of the shoots and roots of transgenic *Arabidopsis* NBR1-overexpressing line revealed differential gene expression in comparison with the nontransgenic wild-type plants [107]. These differentially expressed genes include those involved in responses to stimuli and stress in shoots and those related to translation and formation of ribonucleoprotein complexes in roots [107]. Analysis of interaction network of these differentially expressed genes also indicated a link between NBR1 overexpression and ABA signaling based on the observation that most hubs of this network were associated with ABA signaling [107]. Second, transgenic NBR1-overexpression lines and knockout mutants display phenotypes indicative of fine-tuning of the ABA signaling pathway [107]. These phenotypes include delayed germination, increased number of closed stomata in the NBR1-overexpression lines and increased number of lateral root initiation sites in the knockout mutants [107]. Third, despite altered gene expression associated with ABA responses in the NBR1-overexpressing lines, ABA levels were unchanged in the shoots, suggesting a possible effect of NBR1 on ABA signaling mechanisms [107]. Using bimolecular fluorescence complementation (BiFC), it was demonstrated that NBR1 interacted with three regulatory proteins of ABA pathway (ABI3, ABI4 and ABI5) in planta [107] (Figure 2). NBR1 interaction with ABI5 required its UBA domain but the interactions with ABI3 and ABI4 are independent of the ubiquitin-binding domains [107]. This observation suggests that ABI5, but not ABI3 or ABI4, requires ubiquitination prior to interaction with NBR1. Consistent with the results from BiFC, pull-down assay using recombinant proteins produced in E. coli confirmed the binding of ABI3 and ABI4, but not ABI5, to NBR1, supporting the importance of ubiquitination for ABI5 in its interaction with NBR1. It would be of interest to determine the effect of NBR1 binding on the protein levels of the regulatory proteins of ABA signaling.

## 9. Conclusions and Prospect

As a structural and functional homolog of mammalian p62 and NBR1, plant NBR1 also acts as selective receptor for aggrephagy that targets misfolded proteins and protein aggregates that are generated under a variety of stress conditions. The conserved role of plant NBR1 in selective autophagy likely underlies its critical roles in plant responses to a broad spectrum of biotic and abiotic stresses including heat, drought, salt and oxidative stresses. Plant NBR1 also target specific protein cargoes including heat induced protein chaperones, which paradoxically reduces heat stress memory and compromises tolerance to subsequent heat stress, probably as a mechanism to expedite the recovery of plant growth after the cessation of a heat stress. NBR1 is also increasingly recognized as a critical player in plant antiviral immunity by directly targeting specific viral proteins for degradation in the vacuole. This role of plant NBR1 in plant antiviral defense and counter-defense mechanism through exploitation of plant autophagy by viral pathogens can open new frontiers in the study of the dynamic and complex interactions between plants and microbial pathogens. Through targeting specific target proteins, NBR1-mediated selective autophagy also participates in the modulation of other important processes in plants including stress-induced pexophagy, S nutrient responses and ABA signaling. Despite the progresses, our understanding of NBR1-mediated selective autophagy is still at its infancy. The number of identified cargo proteins for plant NBR1 is still very limited and, as a result, the underlying mechanisms for the broad biological functions of plant NBR1 are still not well understood. Currently there is no comprehensive knowledge about the pathways that regulate the protein levels and activity of plant NBR1 beyond the established fact that the selective autophagy receptor is itself subjected to degradation in the vacuole during autophagy. Animal NBR1 proteins also have novel roles in signaling independent of autophagy and it remains to be determined whether plant NBR1 has similar regulatory roles in signaling in a manner independent of autophagy. A better knowledge about the broad and complex roles of NBR1 will provide new important insights into the molecular basis of plant responses to biotic and abiotic stresses.
